# A Study on the Basic Criteria for Selecting Heterogeneity Parameters of F18-FDG PET Images

**DOI:** 10.1371/journal.pone.0164113

**Published:** 2016-10-13

**Authors:** Attila Forgacs, Hermann Pall Jonsson, Magnus Dahlbom, Freddie Daver, Matthew D. DiFranco, Gabor Opposits, Aron K. Krizsan, Ildiko Garai, Johannes Czernin, Jozsef Varga, Lajos Tron, Laszlo Balkay

**Affiliations:** 1 Scanomed Nuclear Medicine Center, Debrecen, Debrecen, Hungary; 2 Department of Nuclear Medicine, Faculty of Medicine, University of Debrecen, Hungary; 3 Ahmanson Biological Imaging Center, Department of Molecular and Medical Pharmacology, David Geffen School of Medicine at University of California at Los Angeles, California, United States of America; 4 Alfred Mann Institute for Biomedical Engineering, University of Southern California, Los Angeles, California, United States of America; 5 Quantitative Imaging and Medical Physics at Medical University of Vienna, Vienna, Austria; Banner Alzheimer's Institute, UNITED STATES

## Abstract

Textural analysis might give new insights into the quantitative characterization of metabolically active tumors. More than thirty textural parameters have been investigated in former F18-FDG studies already. The purpose of the paper is to declare basic requirements as a selection strategy to identify the most appropriate heterogeneity parameters to measure textural features. Our predefined requirements were: a reliable heterogeneity parameter has to be volume independent, reproducible, and suitable for expressing quantitatively the degree of heterogeneity. Based on this criteria, we compared various suggested measures of homogeneity. A homogeneous cylindrical phantom was measured on three different PET/CT scanners using the commonly used protocol. In addition, a custom-made inhomogeneous tumor insert placed into the NEMA image quality phantom was imaged with a set of acquisition times and several different reconstruction protocols. PET data of 65 patients with proven lung lesions were retrospectively analyzed as well. Four heterogeneity parameters out of 27 were found as the most attractive ones to characterize the textural properties of metabolically active tumors in FDG PET images. These four parameters included Entropy, Contrast, Correlation, and Coefficient of Variation. These parameters were independent of delineated tumor volume (bigger than 25–30 ml), provided reproducible values (relative standard deviation< 10%), and showed high sensitivity to changes in heterogeneity. Phantom measurements are a viable way to test the reliability of heterogeneity parameters that would be of interest to nuclear imaging clinicians.

## Introduction

Medical imaging methods have an increasing role in cancer diagnostics and the assessment of therapy responses. Imaging methods providing lesion quantification information substantially help the evaluation of the efficiency of a chosen treatment. Lesion volume is the simplest parameter that can be determined when using any medical imaging methods. For a more complex characterization of lesions, quantitative measures can be routinely used in case of some imaging methods. Computed Tomography (CT), Magnetic Resonance (MR), Single Photon Emission Computed Tomography (SPECT), and Positron Emission Tomography (PET) are the most frequently used tomographic techniques. When considering these techniques overall, one of the advantages of PET imaging is that it is highly quantitative. The pixel values are directly related to the radiopharmaceutical uptake of the investigated organs. In case of the F^18^-FDG scans, the standardized uptake values (SUV_max_, SUV_mean_, SUV_peak_) are the most frequently calculated quantitative parameters in oncological applications. A large number of articles have been published during the last decade to establish and reveal which SUV values and what cut off levels are the most appropriate for different tumor types in therapy monitoring and staging of cancer patients. Both the volume analysis and the SUV are confounded by the Partial Volume Effect (PVE). This effect is due to the limited spatial resolution and relatively high noise contributions of the PET systems. Therefore, any additional quantitative parameter would improve the prognostic and diagnostic capabilities of PET.

Tumor textural analysis is a new research field of growing interest and might give new insights for the quantitative characterization of tumors [1–10]. It has been determined that heterogeneity of the metabolically active tumor volume is mostly associated with necrosis, hypoxia, cellular proliferation, and angiogenesis. A number of these processes are closely related to cancer aggressiveness and prognosis [11–13]. Accordingly, local and regional texture analysis may provide additional information about the selected tumor tissue. In recent years, more than thirty different textural parameters have been used and investigated in F^18^-FDG studies [14]. However, their clinical utility and reliability are still uncertain. Brooks et al. state that any of the heterogeneity parameters should not be presumed as useful and reliable, and that one should not use them for clinical studies before complete mathematical and methodological standardization analyses have been performed [15–18]. They also demonstrate that the heterogeneity measures could be very sensitive to the volume in the case of small (<45 ml) tumors because of the limited spatial resolution of current PET scanners. Hatt et al. also found similar behavior for volumes above 10 ml, although the provided textural information increases extensively at larger volumes [19]. In addition, Galavis et al. and Jianhua et al. reported large variations for several heterogeneity parameters due to different acquisition and reconstruction parameters [20,21]. Recently, Orlhac et al. [9] performed a comprehensive analysis including 31 textural indices with 3 different human cancer types and revealed that many heterogeneity parameters are somewhat redundant with one another. They also found that the heterogeneity values might depend on the tumor segmentation methods and the number of the predefined grey levels, as well. As a result, they proposed the use of only 4 robust and independent parameters for further investigation: Homogeneity, Entropy, Short-Run Emphasis (SRE), or Zone Length Non-Uniformity.

Phantom measurements could clarify the reliability of any textural characterization, excluding the overall bias caused by biological variability of human studies [14,22–26]. To the best of our knowledge, a limited number of papers deal with phantom-based analysis of textural features. A specially designed phantom was used for a reliability study to calculate different textural indices producing heterogenic activity distribution [27]. Nyflot et al. investigated the effects of stochastic image acquisition noise on the quantitative performance of the heterogeneity parameters with the help of the standard NEMA Image Quality phantom [28]. Orlhac and her coworkers presented work regarding how the resampling approach affects the ability of textural indices, utilizing uniform phantom measurement in addition to patient data [29]. In this study we propose phantom measurements and analysis to help choosing appropriate parameters to quantify tumor heterogeneity with well-defined conditions (reconstruction method, acquisition, minimal volume, segmentation). The following essential criteria were applied for a set of heterogeneity parameters: volume-independence, reproducibility, and quantitative ability to express the degree of heterogeneity. Once establishing this criteria, the selection of the most promising heterogeneity parameters (HePs) out of 27 parameters is analyzed and discussed.

## Methods and Materials

### PET/CT Acquisitions and Reconstructions

Measurements were performed using Philips Gemini TF 64 (institute of author^1^), GE Discovery ST 8 (VUE Point HD) (institute of author^1^) and Siemens Biograph mCT (institute of author^3^) systems. The acquisitions and reconstructions were carried out using the default whole-body FDG-PET protocols for the Philips Gemini TF 64 and GE Discovery ST 8 scanner. On the Siemens Biograph system, a set of reconstructions were applied (see Table 1) using a variety of acquisition times in the range of 60 to 240 sec per bed position, including the factory default protocol.

**Table 1:** Different settings of reconstruction methods for phantom measurements on Siemens Biograph mCT scanner.

Type of Reconstruction	TOF	TrueX	Gauss filter [mm]		Pixel size [mm]	
			4	5	4	3,13
A	-	-	-	+	+	-
B	+	-	-	+	+	-
C	-	-	-	+	-	+
D	-	+	-	+	+	-
E	-	-	+	-	+	-
F	+	+	-	+	+	-

The “+” and “–“denote “yes” or “not” respectively to the application of the indicated reconstruction option.

### Phantoms

**Fig 1:**
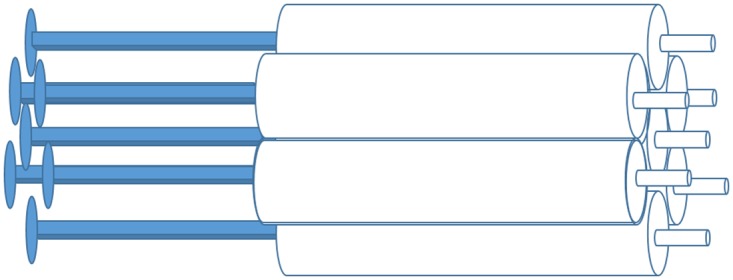
Revolver heterogeneous phantom insert.

A heterogeneous phantom insert (Revolver) was constructed using 7 pieces of 3ml syringes (inner diameter 8.66 mm) arranged in a revolver form (Fig 1). A 20 cm diameter standard cylindrical phantom supplied by the manufacturer was also used to maintain homogeneous activity distribution, designated as the uniform phantom.

### Patient Investigations

In addition to phantom measurements, PET data of 65 patients with confirmed lung lesions were retrospectively analyzed. Histological categorization of these tumors fell beyond our scope since focus was on the limitations of PET imaging. Patients fasted for at least 6 hours before the intravenous administration of 350–400 MBq of 18F-FDG. Blood glucose levels were always under 12 mmol/l. All patients were examined on the Philips Gemini TF scanner. Emission scans began 60 min after injection. Acquisition duration was 60–150 seconds per bed position, depending upon the patient weight. Delineation of lesion volumes was carried out by isocontouring with threshold at SUV = 2.5 g/cm^3^. Low dose CT scans were carried out without oral or intravenous administration of contrast agent. The study was approved by the local ethics committee (Regional And Institutional Ethics Committee, Clinical Center, University Of Debrecen). As the whole patient related data was a retrospectively analyzed, informed consent was not obtained.

### Texture Parameters

All the images were evaluated using the Interview Fusion medical imaging software (Mediso Medical Imaging Systems Ltd., Budapest, Hungary). The computed heterogeneity parameters included Coefficient of Variation and the 26 derived matrix based heterogeneity parameters listed in Table 2. The proper equations are implemented based on Reference [30].

**Table 2:** List of Indices Calculated from Texture Matrices, followed by the short name.

	Name of the heterogeneity parameter (HEP)
Co-occurence matrix based indexes*	• Homogeneity- HOM;
	• Correlation- COR;
	• Entropy-ENT;
	• Contrast-CON
	• Intensity Variability-IV;
Volumetric Zone length statistics	• Zone Percentage- ZP;
	• Size-Zone Variability-SZV;
	• Short Zones Emphasis-SZE;
	• Long Zones Emphasis-LZE;
	• Grey-Level Non-Uniformity-GLNU_Z_
	• Low Grey-Level Zone Emphasis-LGLZE;
	• High Grey-Level Zone Emphasis- HGLZE;
	• Short Zone Low Grey Level Emphasis-SZLGLE;
	• Short Zone High Grey-Level Emphasis- SZHGLE;
	• Long Zone Low Grey Level Emphasis-LZLGLE;
	• Long Zone High Grey-Level Emphasis- LZHGLE;
Volumetric Run Length Statistics	• Run Percentage-RP;
	• Short Run Emphasis-SRE;
	• Long Run Emphasis- LRE;
	• Grey-Level Non-Uniformity- GLNU_R_;
	• Low Grey Level Run Emphasis-LGLRE;
	• High Grey Level Run Emphasis-HGLRE;
	• Short Run Low Grey-Level Emphasis- SRLGLE;
	• Short Run High Grey-Level Emphasis- SRHGLE;
	• Long Run Low Grey-Level Emphasis-LRLGLE;
	• Long Run High Grey-Level Emphasis-LRHGLE

*The co-occurrence type features were calculated for 26 different nearest neighbour connectivity and finally averaged over these directions.

The voxel values of the segmented volume were resampled to yield a finite range of values allowing textural analysis using:

$$V\left(x\right)=\left[2^{s}\frac{I\left(x\right)-\text{min}\left(I\right)}{\text{max}\left(I\right)-\text{min}\left(I\right)+1}\right]$$ (1)

*2*^*S*^ represents the bit depth (in this study 64), *I(x)* is the value of a given voxel of the original image [14].

### Volume dependence

The homogeneous phantom was filled with 5 kBq/ml F-18 activity concentration and imaged according to the routine patient examination protocol on each scanner. Heterogeneity parameter values were calculated using concentric spherical Volume of Interests (VOIs) with volumes ranging from 0.5ml to 1000ml. The calculated parameters were then plotted versus VOI volumes.

### Reproducibility

**Fig 2:**
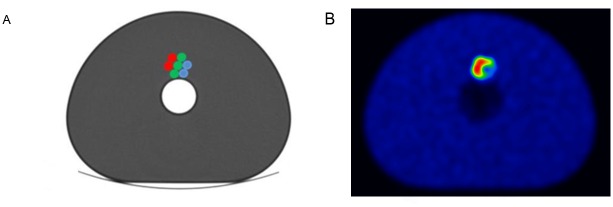
Revolver insert placed in the NEMA IQ Phantom. Illustrative schematic layout, (A) and a representative slice of the attenuation corrected PET image (B).

The reproducibility of the heterogeneity parameters was tested with the help of the heterogeneous phantom insert (Revolver insert) and NEMA IQ phantom. Revolver insert was placed into the NEMA IQ phantom (Fig 2A) filled with 5 kBq/ml F-18 activity concentration, while the syringes contained F-18 activity concentrations of 20 kBq/ml (blue syringes on Fig 2A), 40kBq/ml (green syringes on Fig 2A), and 80 kBq/ml (red syringes on Fig 2A), as displayed on Fig 2. This phantom setup was prepared and measured three times on the Siemens Biograph mCT scanner. Each of the three scan applied all the reconstructions listed in Table 1. in combinations of 60, 120, 180 and 240 sec/bed position.

The high uptake volume imitating a lesion was defined by voxels with SUV>2.5 times the background within a boundary box. Mean value and standard deviation were calculated for each heterogeneity parameter from results of the 3 independent image sets. These calculations were carried out for each combination of acquisition times and reconstruction methods. The ratio of the standard deviation and mean (Coefficient of Variation) was used as the measure of error for the reproducibility of the selected parameter.

### Parameter Sensitivity

Heterogeneity parameter values were determined for patterns with various degrees of heterogeneity. For this purpose, a part of Revolver insert was filled with C-11 solution (80 kBq/ml) and the remaining part of it with F-18 solution (40 kBq/ml). The Revolver insert was surrounded by F-18 water solution (5 kBq/ml) as homogeneous background.

**Fig 3:**
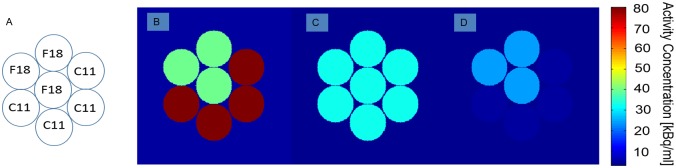
The geometry (A) and the activity distribution within the Revolver insert at (B) t = 0min, (C) t = 45 min, and (D) t = 80 min.

PET scans were performed with 2min acquisitions each followed by a 2 min period of no acquisition. This was repeated over 20 cycles resulting in a total duration of 80 min on the Philips TF 64 scanner. Due to the difference in half-life between C-11 and F-18, the heterogeneity within the insert evolved over time as indicated in Fig 3. The dynamic data included an image at t = 45 min when the activity concentrations are equal for all syringes (Fig 3C). The VOI used for this set of measurements was defined by this image using a 2.5xBg threshold. Reconstruction was performed using the factory default protocol on Philips Gemini TF 64 scanner.

## Results

### Volume Dependence

**Fig 4:**
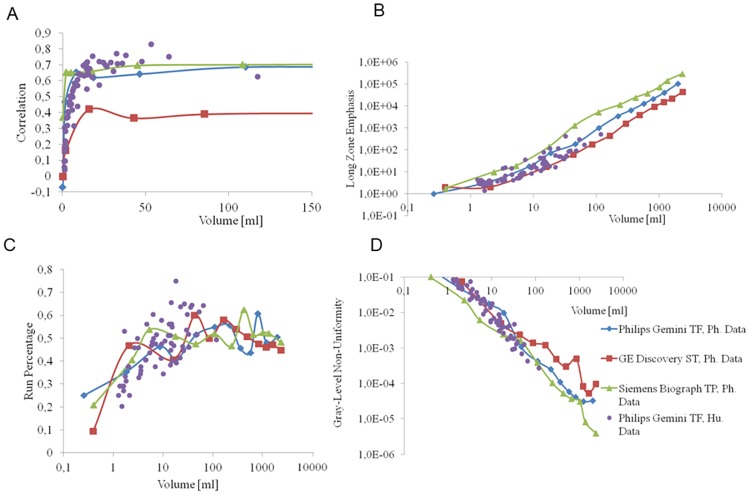
Representative volume dependence of four different HePs. Phantom data points (“Ph.Data”) measured on three different scanners are differentiated by continuous color lines. Individual data points calculated from human lung lesions are displayed as the individual purple dots (“Hu.Data”). The volume dependence of the all investigated parameters can be found in the Supplemental Material.

Plots of the heterogeneity parameters versus the homogeneous volumes indicated that all of the texture parameters are volume dependent. Based on these results, we identified four different kinds of categories (Fig 4). The plots of volume dependence of the all investigated parameters can be found in S1 Fig.

In the first category, the volume dependence of the HeP values were limited to 25–30 ml volume range (Fig 4A). The second and third categories displayed volume dependence in the form of positive and negative slopes in log-log space, respectively (Fig 4B and 4C). The fourth category consisted of parameters which cannot be characterized by a simple function (Fig 4D). Table 3. summarizes the HePs and their categories.

**Table 3:** The Classification of 26 Textural Indices According to the Kind of Dependency of Parameter vs. Volume.

Converging (A)	Positive slope (B)	Random like (C)	Negative slope (D)
ENT, COR, HOM, CON, SZE, LGLZE, SZHGLE, HGLRE,	LZE, LZLGLE, LZHGLE, LRE	HGLZE, SZLGLE, LRLGLE, LRHGLE, RP	SZV, IV, GLNU_Z_, GLNU_R_, LGLRE, SRLGLE, SRHGLE, SRE, ZP,

The Fig 4 plots also demonstrate that the HeP data calculated from the human lung lesions and the homogenous phantom both fall in the similar ranges and follow the same behavior. This result emphasis the relevance and importance of the simple homogenous phantom test to validate any promising HeP candidate.

### Reproducibility

**Fig 5:**
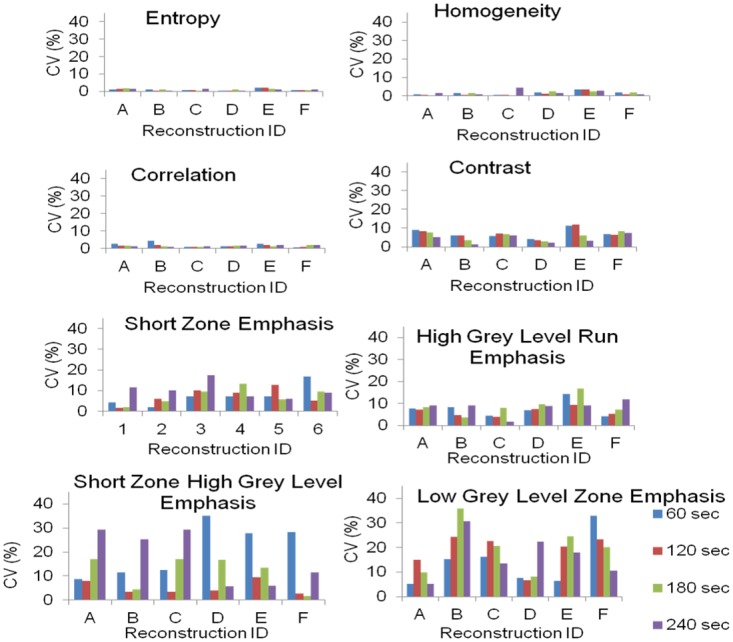
Reproducibility of the 8 remaining HePs (see Table 3, type A), as the function of reconstruction settings (see in Table 1) and acquisition time.

For parameters in the first category (first column in Table 3) the reproducibility errors (CV) were calculated and plotted (Fig 5) based on the heterogeneous phantom data from 3 independent scans. For further analysis we considered only those parameters where the CV values were less than 10% for almost all of the acquisition times and reconstruction settings. These parameters included Entropy, Homogeneity, Correlation, and Contrast.

### Coefficient of Variation

**Fig 6:**
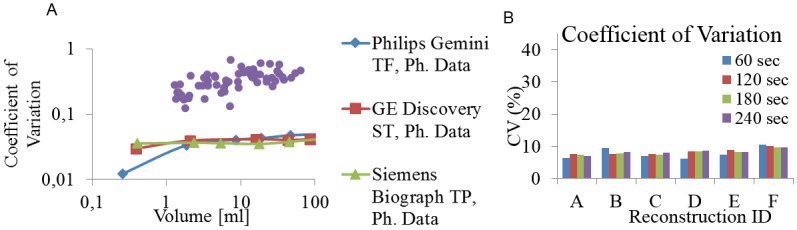
Volume dependence (A) and reproducibility (B) of the Coefficient of Variation parameter.

Calculation of the Coefficient of Variation parameter differs from HePs calculated using textural matrices. It does not require resampling and further matrix calculation; it is the ratio of deviation over mean. This difference in calculation method may account for the fact that the numerical values of Coefficient of Variation parameter evaluated from reconstructed human lung images differ in a magnitude from data derived from homogeneous phantom measurements (Fig 6A). Based on the results on Fig 6. the Coefficient of Variation parameter was included to the list of converging and reproducible parameters.

### Parameter Sensitivity

**Fig 7:**
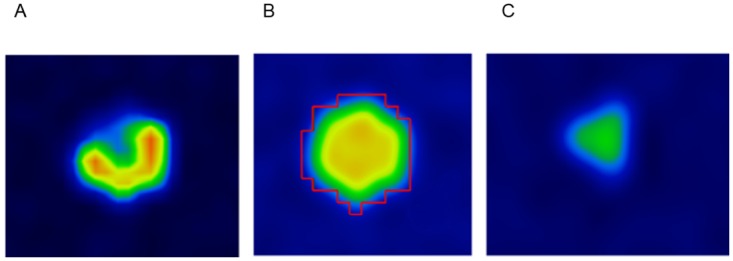
An axial slice of the image of Revolver insert phantom inserted into a homogeneous environment reconstructed from data of 2 min acquisition time beginning at t = 0 (A), t = 45 min (B), and t = 80 min (C). Boundary of the applied VOI is also displayed on panel b.

**Fig 8:**
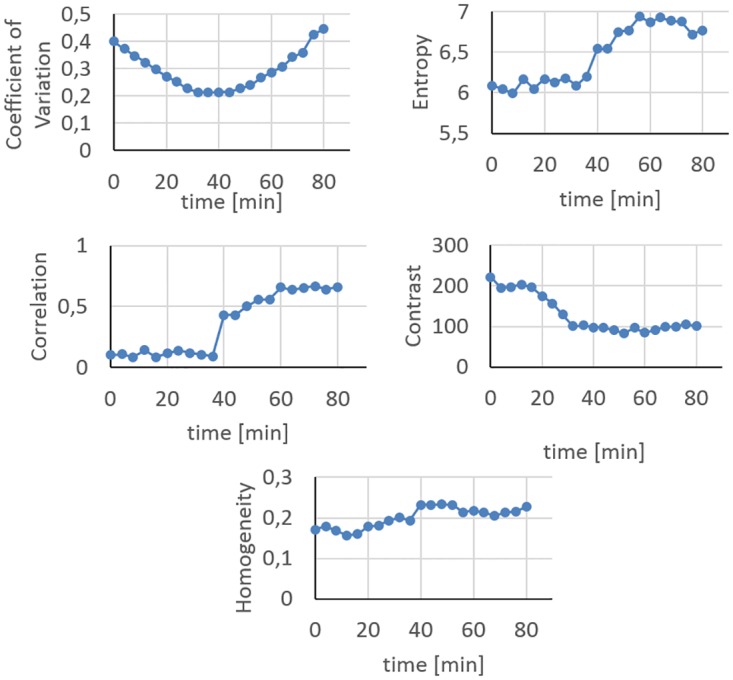
Time dependence of the heterogeneity parameters. Parameter values belonging to different time points are assigned to different textural patterns.

At t = 0 min, the syringes filled with C-11 are far more active than those filled with F-18 as seen in Fig 7A. At t = 45 min, the activity concentrations of the syringes are relatively close resulting in a fairly even activity distribution throughout all syringes (Fig 7B). The boundary of the VOI applied for HeP calculations in this dynamic analysis is displayed on Fig 7B as well. At t = 80 min, the relatively high activity of the F-18 compared to the C-11 results in the image shown in Fig 7C. The heterogeneity parameters were calculated for the different time points, and are plotted on Fig 8.

## Discussion

The main objective of this work was to quantitatively evaluate the reliability of a given set of heterogeneity parameters proposed for PET investigations. The heterogeneity of intratumoral radiopharmaceutical uptake has been suggested as a new quantitative measure and has been intensively studied in a number of recent communications [2,4,5,8,31–36]. These parameters allow the quantification of intratumoral activity patterns that might reflect the metabolic and pathological state of the cancer. On the other hand, these parameters might help to predict the response of a chosen therapy for the patient. An increasing number of tumor heterogeneity studies have been published recently, particularly within the last two years. Review articles of this new field have been also released [1,3,5,37,38], supporting the growing interest of the heterogeneity measurements within the special field of cancer research. Moreover, any imaging based heterogeneity evaluation could lead to more personalized therapy.

Despite all of this great interest, a comprehensive verification of any of the heterogeneity parameters is still missing. Most articles present data about the unreliability of heterogeneity parameters. This unreliability is largely attributed to the lack of rigorous analysis of the effects of tumor volume, spatial resolution, reproducibility and image bit depth on these parameters [17,39]. The accuracy of the segmentation methods varies among different algorithms and also leads to increased reproducibility error [25,26]. To eliminate this variability, we chose the most robust segmentation method: the SUV threshold based segmentation. However, tumor delineation method of choice is a confounding issue, because different groups prefer different approaches. In addition, the defined volume that the texture indices are calculated within may influence the values [9]. More than 40 textural parameters have been proposed by different groups, but only a limited number of studies have clarified the differences among them, and tried to analyze the reliability and robustness of these parameters by applying sophisticated mathematical methods [9,15,34,40,41]. The comparative and follow up human studies have limited applicability for this purpose, because the biological and intersubjective variability may distort the basic characteristics of the selected heterogeneity parameter. In this work, we propose phantom-based methodologies to analyze the capability and reliability of textural parameters used with PET imaging. The tumor volume has to be large enough in order to reflect reliable heterogeneity. Heterogeneity parameters are the results of statistical calculations from segmented voxels; these voxels affect each other due to the limited resolution of the system (i.e. point-spread function). When observing the behavior of a given HeP, the same type of volume dependence was observed in our phantom analysis (Fig 4) and confirmed by our retrospective lung image data. Based on these results we classified the 27 heterogeneity parameters into four clearly differentiated groups (Table 3). Due to their unpredictable volume dependence, the parameters categorized within the random-like group cannot offer useful characterization of texture. A larger number of HeP candidates show volume dependence similar to that displayed on Fig 4B and 4D. These parameters should be excluded from the further analysis as inadequate parameters. It can be strictly stated that well-performing heterogeneity parameters should not show dependence on the volume of the segmented region defined within the boundaries of a homogeneous phantom. Orlhac et. al. [9] reported similar results based on patient data. They have found 10 or 19 parameters out of the investigated 31 as unsuitable if the volume correlation limit was set to |r|>0.8 or |r|>0.6, respectively. Based on our findings, 9 parameters out of the 27 showed volume independency above a minimal volume (25–30 ml; Table 3, Fig 4A). In addition, the human lung lesion data underscores the relevance of the phantom data, since the same behavior and range can be observed for both data sets in Fig 4. Brooks et all. examined the effect of small tumor volumes on the calculation of heterogeneity, using the combination of probability theory and clinical 18-FDG-PET data. They conclude that the heterogeneity parameters of tumor volumes below 45ml can biased [15]. Reproducibility error was calculated for the following 9 well-performing parameters: Entropy, Contrast, Correlation, Homogeneity, Short Zone Emphasis, High Grey Level Run Emphasis, Short Zone High Grey Level Emphasis, Low Grey Level Zone Emphasis and Coefficient of Variation (Figs 5 and 6B). The reproducibility error of Entropy, Homogeneity and Correlation remained under 5% for each imaging protocol used, confirming their acceptable reliability. The error of Coefficient of Variation and Contrast parameters can be reduced below 10% by adequate selections of acquisition time and reconstruction method. The SZE, HGLRE, SZHGLE and the LGLZE were found to be less attractive parameters, and were excluded from further analysis because their error of reproducibility was higher than 10% in more than half of the imaging scenarios. PVE may quantitatively effects the voxel values, especially in the case of small patterns comparable to the spatial resolution. The PVE related behavior can be observed in Fig 4A, where in the range of small volumes (< 25-30ml) the HeP was highly correlated with the volume. In addition, we also present PVE related data in Fig 5, where the reproducibility was investigated as the function of the reconstruction method and the acquisition time, since D and F reconstruction methods applied PVE correction (the so-called ‘TrueX’ option, see Table 1). However the TrueX option did not have great impact on neither the absolute value (S2 Fig) nor the reproducibility of the parameters.

We also constructed a dynamically changing texture by means of a dual isotope phantom. This phantom included separate syringes for F-18 and C-11 isotope and was used to investigate the sensitivity of the remaining HePs (Fig 7). As a result, an initially heterogeneous activity concentration pattern dominated by the C-11 is later transformed to an F-18 dominated heterogeneous pattern, across an equilibrium point. In this equilibrium point (at t = 45min) the C-11 and F-18 activity distribution is equal, producing a fully homogeneous uptake pattern. Fig 8 demonstrates how the Entropy, Coefficient of Variation, Homogeneity, Correlation and Contrast differ in sensitivity and tendency.

The Coefficient of Variation parameter has two maxima according to the initial and the final heterogeneous uptake, and a minimum point at the equilibrium phase. Correlation has the highest sensitivity since the values are changing on a wide scale from 0.1 to 0.7. However, correlation, Entropy and Contrast moderately distinguish between the heterogeneous and homogeneous state. The Homogeneity parameter is a less sensitive parameter since it poorly reflects the degree of heterogeneity. Therefore, it was excluded from the more reliable parameters. The remaining parameters to characterize the textural properties of metabolically active tumors in FDG PET scans which fulfill our criteria are the Entropy, Contrast, Correlation and Coefficient of Variation. These four parameters were selected from 27 candidates, however our selection strategy can be easily applied for any currently existing or newly introduced HeP, as well. Since our selection process was focused on physical validation, the investigation of the inherent prognostic value reflected by the validated parameters needs further human studies with multiple patient cohorts.

Although insightful, one of the primary limitations of this study is the lack of validation regarding the software used for the HeP calculations. In addition, several previously published studies used custom-written software without validation or comparison of the calculated HEP values with other software. Adequate phantom measurements could assist in this validation and comparison task for the whole imaging procedure including the acquisition, reconstruction, segmentation and HeP evaluation.

## Conclusions

The purpose of the paper was to clearly define the criteria used to identify the most appropriate heterogeneity parameters to measure textural features. Based on this criteria, we compared various suggested measures of homogeneity. Our predefined criteria were: a reliable heterogeneity parameter has to be volume independent, reproducible and suitable to express quantitatively the degree of heterogeneity. Detailed phantom measurements and evaluation have been described to reveal the reliability of any existing or newly emerging parameters used to measure textural properties of delineated regions defined on reconstructed PET images. Only four out of the 27 selected textural parameters fully met our criteria. These parameters were the Coefficient of Variation, Contrast, Correlation and Entropy. By using appropriate reconstruction methods, these parameters can provide reproducible values within 10 percent error in tumor volumes > 25–30 ml. Our sensitivity study concluded that these four selected parameters have different levels of robustness to measure the degree of heterogeneity.

## Supporting Information

S1 Fig Volume dependence of all investigated HePs. (PDF)

S2 Fig The real value of the four finally suggested HePs as the function of reconstruction settings (see in Table 1) and acquisition time. (TIF)
