# Supplementary figures and images for: A Study on the Basic Criteria for Selecting Heterogeneity Parameters of F18-FDG PET Images
Source: PLoS One. 2016 Oct 13;11(10):e0164113. doi: 10.1371/journal.pone.0164113 (PMC5063296; doi:10.1371/journal.pone.0164113)

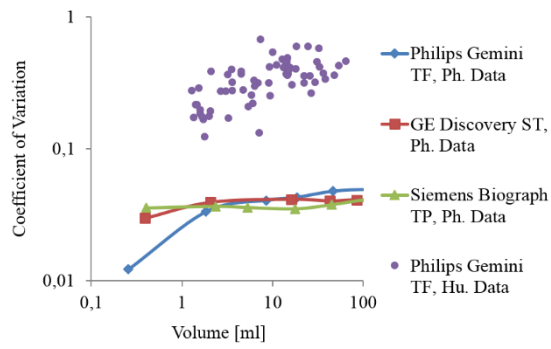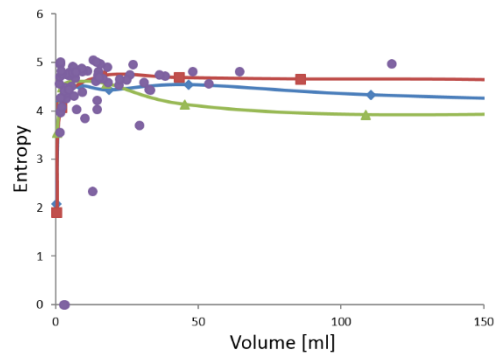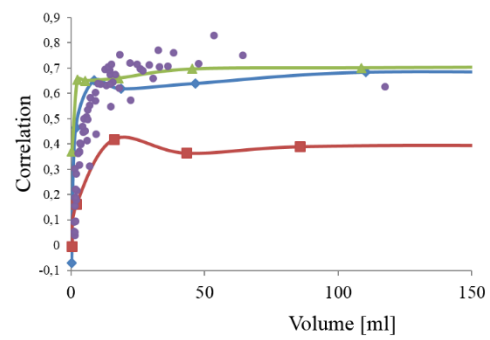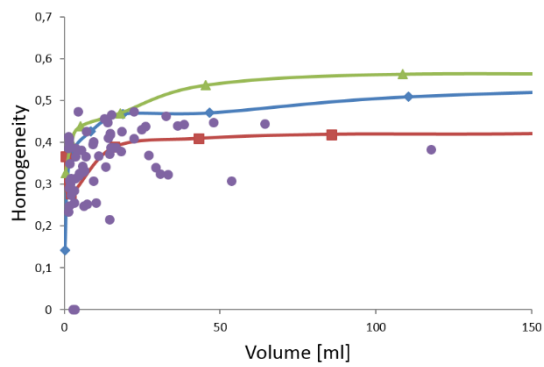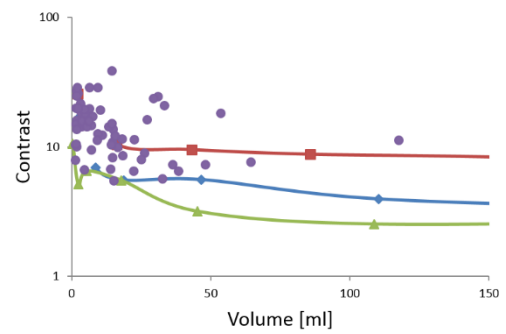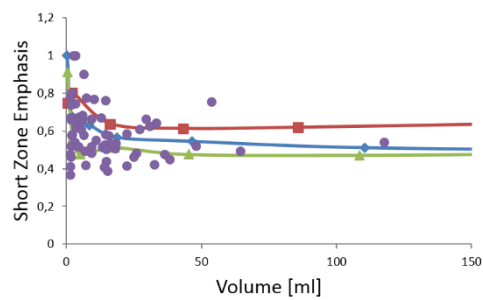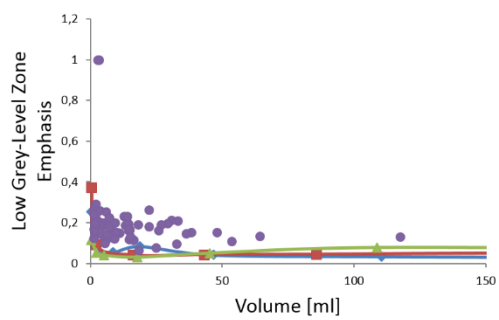

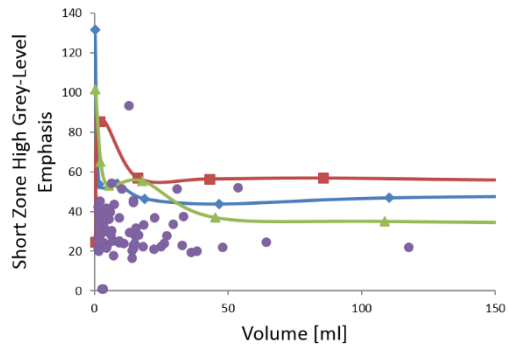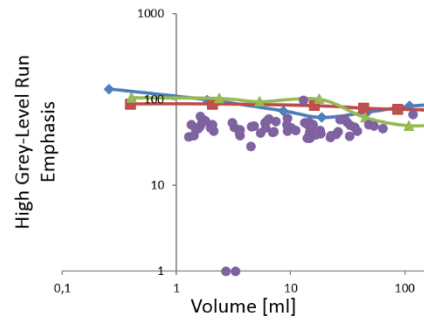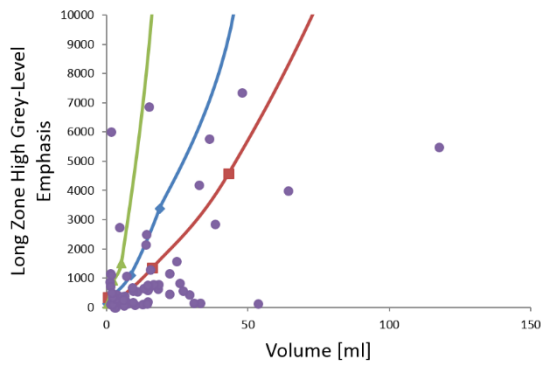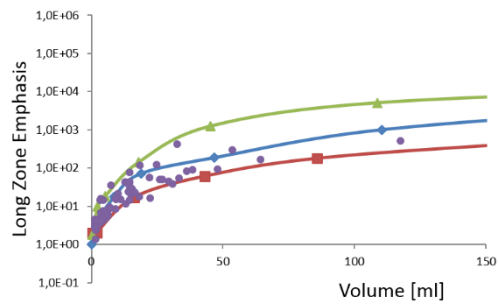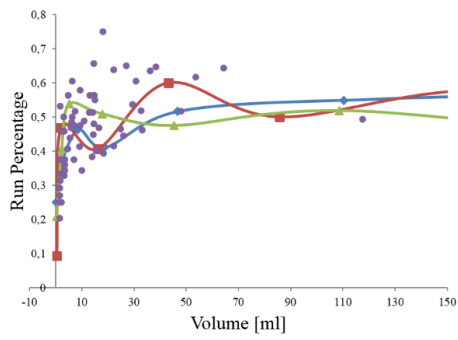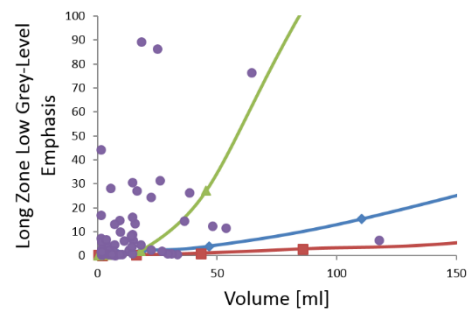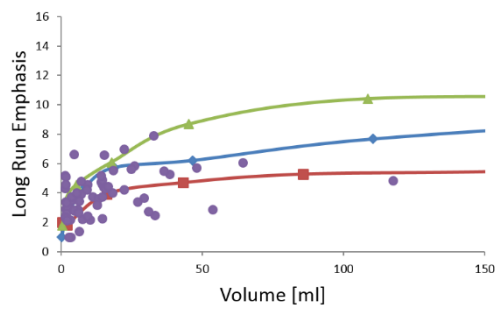

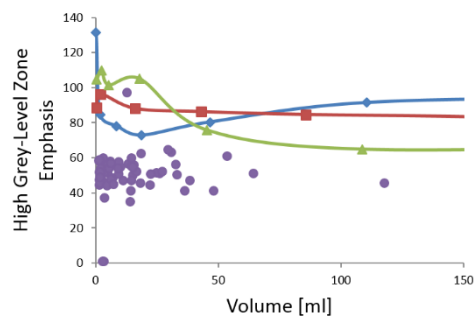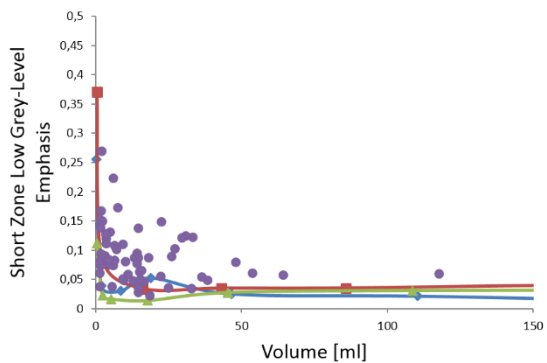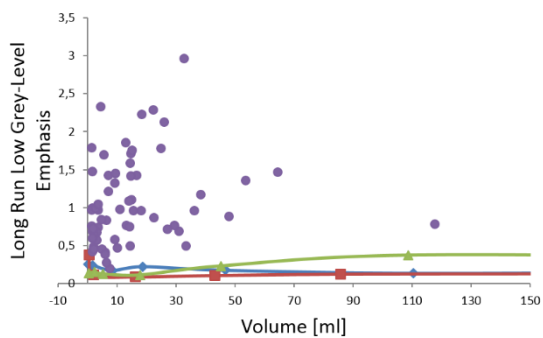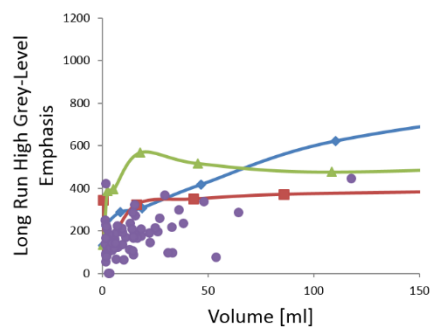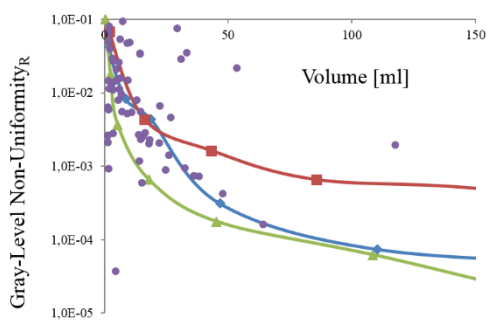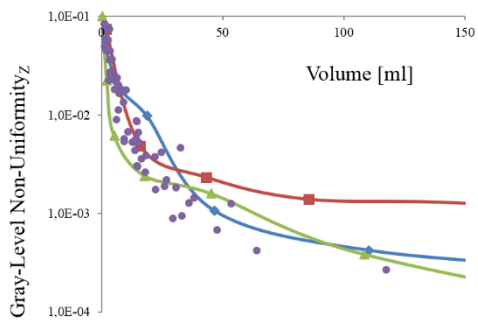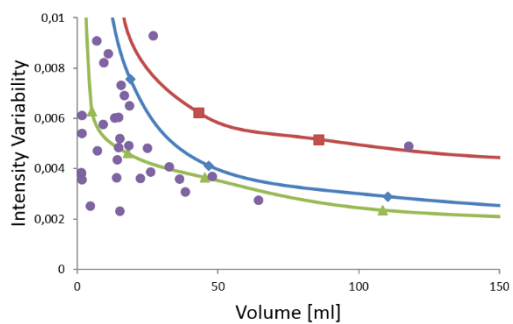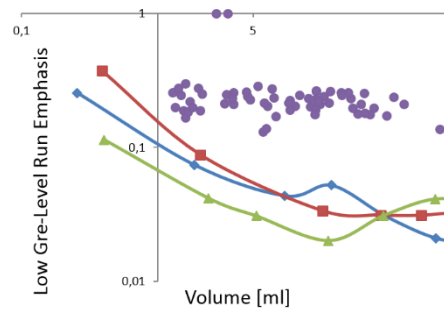

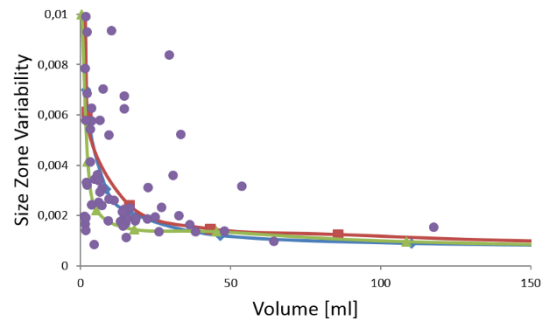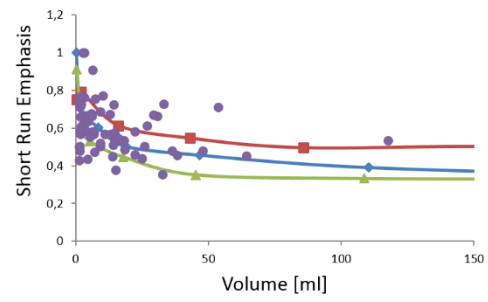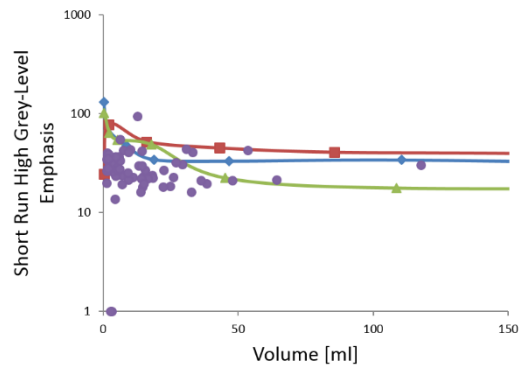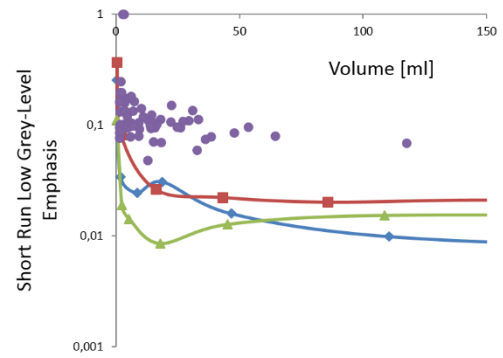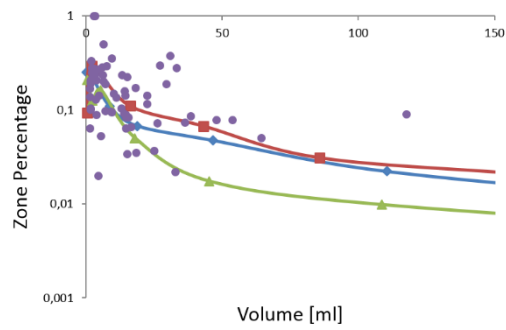

Supplement: S1 Fig — (PDF) [file pone.0164113.s001.pdf]

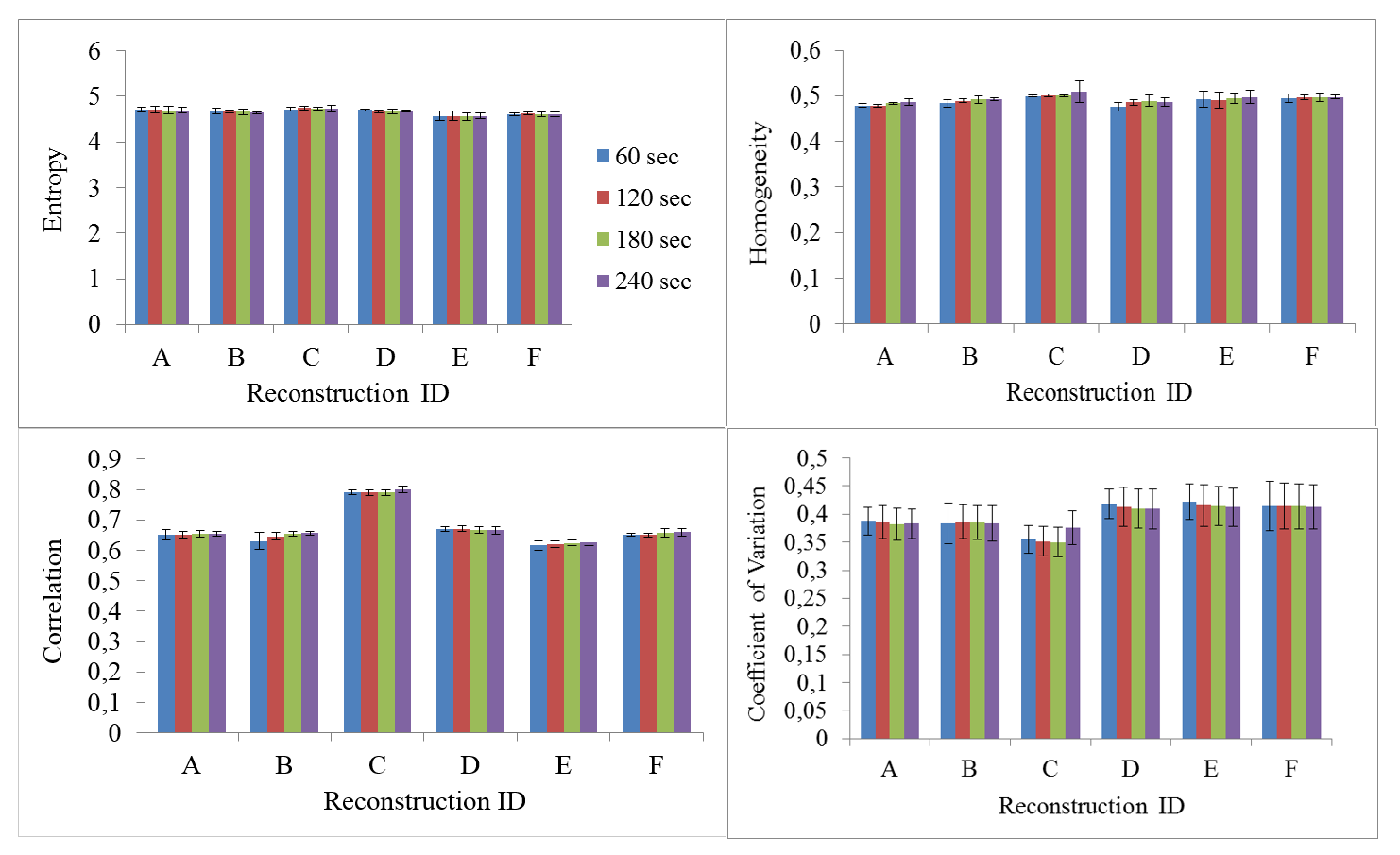

Supplement: S2 Fig — (TIF) [file pone.0164113.s002.tif]
